# ScholarCitation: Chinese Scholar Citation Analysis Based on ScholarSpace in the Field of Computer Science

**DOI:** 10.3389/fdata.2019.00041

**Published:** 2019-11-26

**Authors:** Hanting Su, Zhuoya Fan, Chen Cao, Yi Zhang, Shuo Wang, Xiaofeng Meng

**Affiliations:** Web and Mobile Data Management Lab, Information School, Renmin University of China, Beijing, China

**Keywords:** Chinese literature, citation analysis, ScholarSpace, computer science, Chinese academic data

## Abstract

Citation analysis is one of the most commonly used methods in academic assessments. Up to now, most of academic assessments are based on English literature, ignoring the fact that the role of Chinese papers in academic assessments has become increasingly indispensable. Therefore, to give full play to the role of Chinese literature in academic assessments is an urgent task of current academic circle. Based on Chinese academic data from ScholarSpace, i.e., 82826 Chinese computer science journal papers, we conduct a comprehensive assessment of academic influence from the perspectives of fields, journals and institutions, in order to achieve a better understanding of the development of Chinese computer literature in the past 60 years. We find that Chinese scholars tend to cite papers in English, discover evolution trend of fields, journals and institutions, and call on journals, institutions, and scholars to strengthen their cooperation.

## Introduction

The progress of scientific research is closely related to academic inheritance. Only by standing on the shoulders of predecessors can the younger generation see further. Citation is a form of academic inheritance. Through citation analysis, scholars can understand the development of academic fields and draw lessons from classical research ideas, thus gaining new academic achievements. Therefore, citation analysis is one of the most commonly used methods in academic assessments.

Up to now, most of academic assessments are based on databases that mainly consist of English literature, such as DBLP^1^ (Reitz and Hoffmann, 2010; Song et al., 2014), Scopus^2^ (Leydesdorff et al., 2010; Li et al., 2010; Kousha et al., 2011), Web of Science^3^ (Ellegaard and Wallin, 2015; Marx and Bornmann, 2015; Calma and Davies, 2016; Zhao, 2017) as well as Microsoft Academic^4^ (Harzing, 2016; Effendy and Yap, 2017; Hug et al., 2017). For instance, although Scopus includes a collection of non-English journals, they are still underrepresented, constituting about only 15% of the total number of included journals (de Moya-Anegón et al., 2007). In Web of Science, English-language journals are overrepresented to the detriment of other languages (Mongeon and Paul-Hus, 2016). The situation mentioned above leads to a phenomenon that the more English papers a scholar published and the more citations he received, the higher his academic influence is considered.

However, this assessment method is not entirely suitable for Chinese scholars, since a considerable number of them writes in their native language, and there are many excellent works in Chinese papers [According to (Orduña-Malea and López-Cózar, 2014), Chinese journals prominently occupy second place in the h-index average journal ranking in April and November 2012]. Therefore, it is necessary to pay more attention to Chinese papers in academic assessments. Although some databases such as Google Scholar include a considerable number of Chinese literature (Orduña-Malea and López-Cózar, 2014), related citation analyses are based on citations between both Chinese and English literature. In this case, we aim to implement an academic assessment based on Chinese literature through citation analysis, limiting citations to those between selected Chinese journals, so as to see something that has always been neglected and make our own voice heard.

Our main contributions are listed below:

- Firstly, we implement an academic assessment based on Chinese literature in the field of computer science through citation analysis in the past 60 years.
- Secondly, our citation analysis limits citations to those between selected Chinese journals and reveals the preference of Chinese scholars when citing.
- Thirdly, we introduce innovative indicators to enrich classic citation analysis in both transverse and vertical aspects, and use the algorithm of CSRankings to better calculate the number of citations, which can be applied to similar datasets.

This paper is arranged as follows. Section related work will introduce related work. Section data description will introduce our dataset. Section Indicator Definition will give the definitions of evaluation indicators we designed. The details of citation analysis will be shown in section Citation Analysis. Finally, we will conclude this paper in section conclusion.

## Related Work

### Indicators for Citations

In the light of indicators for citations, previous works have mainly explored impact factor (Institute for Scientific Information., 2002), SJR (SCImago Journal Rank) (SCImago, 2007) and GSM (Google's Scholar Metrics) (Jacsó, 2012).

The impact factor of a certain year Y is calculated by dividing the number of papers published in the two previous years by the number of times papers published in the two previous years were cited during year Y. Although commonly applied, it has weaknesses due to lack of quality assessment for citations (Dellavalle et al., 2007), poor comparability between different domains of interest per journal (Postma et al., 2007) and the possibility of being manipulated by using self-citations, publishing relatively many review articles and limiting the number of articles included (Fassoulaki et al., 2000; Siebelt et al., 2010).

To tackle the problems above, PageRank-like algorithms were designed by assigning weights to different citations (Page et al., 1999; Bergstrom, 2007; SCImago, 2007), among which the most famous one is SJR (SCImago, 2007), applying the PageRank algorithm on the Scopus database, so that citations by more prestigious journals would have more influence compared to other journals (Brown, 2011).

Besides, GSM also provides an indicator, the H-index for the number of citations. A scientist has index h if h of his/her N papers have at least h citations each, and the other (N-h) papers have fewer than h citations each (Bornmann and Daniel, 2005). However, as its maximum value is limited by the total number of papers published by a journal, H-index favors the most productive ones (Delgado-López-Cózar and Cabezas-Clavijo, 2013), which makes it unfair for those journals with a relatively smaller volume but higher quality. In addition, the H-index suffers from low resolution, which means that it is common for a group of researchers to have an identical H-index (Zhang, 2009).

Inspired by SJR and CSRankings^5^, we design a devised version of calculating citations, which will be described in detail in section indicator definition. Similar to SJR, we assign different weights to different citations by splitting citations of one paper evenly to its authors. The difference between our method and SJR is that we don't assign different weights to different journals, while SJR weighs the importance of a journal by its number of citations. Our method is more appropriate to this research for these reasons: firstly, our dataset only includes citations from Chinese journals to Chinese journals, so the number of citations of a journal, especially journals receiving considerable citations from literature in other languages, doesn't necessarily represents its quality; secondly, since journals included in our dataset are carefully selected, the gap between qualities of different journals is relatively small.

Apart from number of citations, we also design rich indicators to provide a more comprehensive citation analysis, which is introduced in detail in sections indicator definition and citation analysis.

### Datasets for Citation Analysis

Table 1 shows the comparison of datasets used for citation analysis in the field of computer science.

**Table 1:** Datasets used for citation analysis in the field of computer science*.

**Database**	**Proportion of English journals**	**Proportion of Chinese journals**	**Starting time**	**Sub-fields available**
Web of Science(core collection)^1^	99.48%	0.00%	1900	Yes
DBLP^2^	100.00%	0.00%	1936	No
Scopus^3^	79.82%	1.46%	1996	Yes
**Database**	**Proportion of English papers**	**Proportion of Chinese papers**	**Starting time**	**Sub-fields available**
Google Scholar^4^	23.86%	42.54%	Unknown	Yes
CSCD^5^	1.66%	89.17%	1989	No
ScholarSpace^6^	0.00%	100.00%	1960	Yes

1 *Updated in 2018, data collected from: https://jcr.clarivate.com/JCRLandingPageAction.action, journals in multiple languages are excluded*.

2 *Updated in April 2019, data collected from: https://dblp.org/xml/release/*.

3 *Updated in May 2019, data collected from: https://www.elsevier.com/__data/assets/excel_doc/0015/91122/ext_list_May_2019.xlsx*.

4 *According to (Orduña-Malea and López-Cózar, 2014), up to March 2013, 42.54% of the records included in Google Scholar are from China, 23.86% are from USA and UK*.

5 *Updated in 2019, data collected through advanced search of Web of Science*.

6 Updated in January 2019, data collected from: http://cdblp.ruc.edu.cn/computer/. *Statistics are limited to the field of computer science.

As is shown above, our dataset differs from traditional datasets in aspects of language coverage, time coverage and sub-field information, thus enables us to implement a both transversely and vertically deep analysis of the development of Chinese computer literature, including analysis on journals, sub-fields and institutions as well as cross analysis. Most importantly, our dataset limits citations to those between selected Chinese journals, while others include citations between both Chinese and English (and other languages) literature.

## Data Description

ScholarSpace^6^ (Liu et al., 2009; Chen et al., 2011) is a Chinese academic information integration system. In order to distinguish publications written by authors with identical names, ScholarSpace employs an improved version of GHOST (Fan et al., 2008) algorithm for name disambiguation. Since its launch in 2008, the ScholarSpace (C-DBLP) system has continued to provide multi-disciplinary Chinese literature query, analysis and guided reading services for 10 years. With the accumulation of data, the original method of displaying knowledge can no longer meet the needs of users. For this reason, Web and Mobile Data Management (WAMDM) Lab has developed a series of simple and practical sub-systems to explore new knowledge from different angles and provide users with various services.

As a subsystem of ScholarSpace, our dataset, ScholarCitation has obtained 71,221 citation relationships of 82,826 journal papers from top 11 Chinese computer science journals, i.e., *Chinese Journal of Computers(CJC), Journal of Computer Research and Development(JCRD), Scientia Sinica (Informationis)(SS-I), Journal of Software(JS), Journal of Computer-Aided Design* & *Computer Graphics(JCAD*&*CG), Acta Electronica Sinica(AES), Journal of Chinese Information Processing(JCIP), Journal of Frontiers of Computer Science and Technology(JFCST), Journal of Chinese Computer Systems(JCCS), Journal of Image and Graphics(JIG)*, as well as *Computer Science(CS)*, including the 10 sub-fields of computer science defined by CCF (China Computer Federation), i.e., Computer Network and Communication(Network), Information Security and Privacy Protection(Security), Chinese Information Processing and Information Retrieval(Info), Computer Theory(Theory), Graphics and Human-Computer Interaction(Graphics & HCI), Software Engineering/System Software/Program Designing(Software), Database System and Data Mining(Database), Architecture and High Performance Computing(Architecture), Artificial Intelligence and Robotics(AI), as well as Intersection and Synthesis(Intersection).

**Figure 1:**
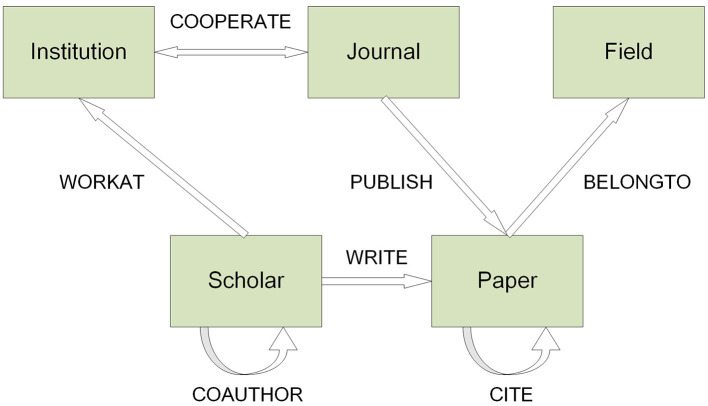
Ontology of the dataset.

The ontology of our dataset is shown in Figure 1. As is shown in Figure 1, the dataset contains 5 entities, i.e., institutions, journals, fields, scholars, and papers. As for relations, it contains 7 relations, i.e., the CITE relation among papers, the WRITE relation between scholars and papers, the WORKAT relation between scholars and institutions, the COAUTHOR relation among scholars, the COOPERATE relation between institutions and journals, the PUBLISH relation between journals and papers, as well as the BELONGTO relation between papers and fields. This paper will focus on fields, journals and institutions based on the 71,221 CITE relations of 82,826 journal papers.

## Indicator Definition

For ease of explanation, the 82,826 journal papers collected by ScholarCitation will be referred to as *target papers* in the following analysis. We define a series of evaluation indicators for three types of entities: fields, journals, and academic institutions, including the number of papers, the number of self-citations, the number of other-citations, the number of other-references, the number of citations, the number of references, self-citation rate, citation rate, and reference rate. The definitions of these indicators are shown in Table 2.

**Table 2:** Indicator definition.

**Indicator**	**Definition**	**Example/Calculation method**
Number of papers P(E, T)	The total number of target papers published by entity E in a period T	P(CAS, [2009, 2018]) means the total number of target papers published by CAS in the last 10 years
Number of self-citations C_S− S_(E, T)	The total number of times that entity E cited its own published target papers in a period T	C_S− S_(CAS, [2009, 2018]) means the total number of times that CAS cited its own published target papers in the last 10 years
Number of other-citations C_O− S_(E, T)	The total number of times that the target papers published by entity E were cited by other entities in a period T	C_O− S_(CAS, [2009, 2018]) means the total number of times that other institutions cited the target papers published by CAS in the last 10 years
Number of citations Ced(E, T)	The total number of times that the target papers published by entity E were cited by all entities (itself and other entities) in a period T	Ced(E, T) = C_O− S_(E, T) + C_S− S_(E, T)
Number of other-references C_S− O_(E, T)	The total number of times that the entity E cited target papers published by other entities in a period T	C_S− O_(CAS, [2009, 2018]) means the total number of times that CAS cited the target papers published by other institutions
Number of references C(E, T)	The total number of times that the entity E cited target papers published by all entities (itself and other entities) in a period T	C(E, T) = C_S− O_(E, T) + C_S− S_(E, T)
Self-citation rate CR_S− S_(E, T)	The number of self-citations of entity E per paper in a period T	$\text{C}{\text{R}}_{\text{S}-\text{S}}\left(\text{E},\text{T}\right)=\frac{{\text{C}}_{\text{S}-\text{S}}\left(\text{E},\text{T}\right)}{\text{P}\left(\text{E},\text{T}\right)}$
Citation rate CedR(E, T)	The number of citations of entity E per paper in a period T	$\text{CedR}\left(\text{E},\text{T}\right)=\frac{\text{Ced}\left(\text{E},\text{T}\right)}{\text{P}\left(\text{E},\text{T}\right)}$
Reference rate CR(E, T)	The number of references of entity E per paper in time T	$\text{CR}\left(\text{E},\text{T}\right)=\frac{\text{C}\left(\text{E},\text{T}\right)}{\text{P}\left(\text{E},\text{T}\right)}$

Further explanation of these indicators is provided below.

First, the number of papers *P*(*E, T*) means the total number of target papers published by entity E in a period T. For example, assuming that Chinese Academy of Sciences(CAS) published 1000 papers between 2009 and 2018, then *P*(*CAS*, [2009, 2018]) is 1000.

Second, the number of self-citations *C*_*S*−*S*_(*E, T*) means the total number of times that entity E cited its own published target papers in a period T, and the meaning of self-citation rate *CR*_*S*−*S*_(*E, T*) is the number of self-citations of entity E per paper in a period T. For example, assuming that CAS published 4000 papers and cited itself 2000 times between 2009 and 2018, then *C*_*S*−*S*_(*CAS*, [2009, 2018]) is 2000, and *CR*_*S*−*S*_(*CAS*, [2009, 2018]) is the ratio of 2000 to 4000, i.e., 0.5.

Third, the number of other-citations *C*_*O*−*S*_(*E, T*) means the total number of times that the target papers published by entity E were cited by other entities in a period T. For example, assuming that CAS was cited by other institutions 3000 times between 2009 and 2018, then *C*_*O*−*S*_(*CAS*, [2009, 2018]) is 3000.

Fourth, the number of citations *Ced*(*E, T*) means the total number of times that the target papers published by entity E were cited by all entities (itself and other entities) in a period T, and the meaning of citation rate *CedR*(*E, T*) is the number of citations of entity E per paper in a period T. For example, if *C*_*S*−*S*_(*CAS*, [2009, 2018]) is 2,000 and *C*_*O*−*S*_(*CAS*, [2009, 2018]) is 3,000, then *Ced*(*CAS*, [2009, 2018]) is the sum of 2,000 and 3,000, i.e., 5,000. At the same time, if *P*(*CAS*, [2009, 2018]) is 1,000, then *CedR*(*CAS*, [2009, 2018]) is the ratio of 5,000 to 1,000, i.e., 5.

Fifth, the number of other-references *C*_*S*−*O*_(*E, T*) means the total number of times that the entity E cited target papers published by other entities in a period T. For example, assuming that CAS cited other institutions 4,000 times between 2009 and 2018, then *C*_*S*−*O*_(*CAS*, [2009, 2018]) is 4,000.

Sixth, the number of references *C*(*E, T*) means the total number of times that the entity E cited target papers published by all entities (itself and other entities) in a period T, and the meaning of reference rate *CR*(*E, T*) is the number of references of entity E per paper in time T. For example, if *C*_*S*−*O*_(*CAS*, [2009, 2018]) is 4,000 and *C*_*S*−*S*_(*CAS*, [2009, 2018]) is 2,000, then *C*(*CAS*, [2009, 2018]) is the sum of 4,000 and 2,000, i.e., 6,000. At the same time, if *P*(*CAS*, [2009, 2018]) is 1,000, then *CR*(*Academy of Sciences*, [2009, 2018]) is the ratio of 6,000 to 1,000, i.e., 6.

In addition, we define citation score *CS*(*E, T*) for entities of institutions. The meaning of *CS*(*E, T*) is the sum of citation scores of entity E in a period T. The calculation method of this indicator is based on the CSRankings^7^ methodology. Specifically, we distribute the citations of each paper equally to each author, thus obtaining the citation score of the scholar on the corresponding paper. The sum of citation scores obtained by a scholar on all papers published in a given year is denoted as the scholar's total citation score in that year. The annual citation score of an institution is the sum of all its scholars' total citation score in that year. This calculation method takes into account that a paper is the contribution of all the authors. For example, one scholar wrote a paper by himself, while another scholar wrote a paper with 9 collaborators. If both of the two papers have 10 citations, we cannot assume that the two scholars have the same contribution on the two papers.

## Citation Analysis

This section will first give an overview of our citation analysis in section overview of citation analysis, then conduct the citation analysis based on fields, journals and institutions in section fields-based citation analysis, section journals-based citation analysis, and section journals-based citation analysis respectively. In section cross citation analysis, we will finish cross citation analysis.

### Overview of Citation Analysis

#### Total Self-Citation Rate

By 2018, ScholarCitation has collected 71,221 self-citation relationships of 82,826 target papers. Therefore, the self-citation rate of ScholarCitation is 0.86. However, from the official website of Scimagojr^8^, it shows that based on Chinese journals(account for 1.46% of journals in Scopus and include 27.27% of journals in ScholarSpace) and English journals(account for 79.82% of journals in Scopus) included in Scopus database, the self-citation rate of Chinese and English papers published by Chinese scholars in the field of computer science, is 2.60 by 2018.

By comparison, in the field of computer science, the self-citation rate of Chinese and English journal papers published by Chinese scholars is much higher than that of Chinese journal papers. Therefore, **merely considering self-citation, Chinese scholars prefer to cite English papers**.

#### Trend of Development

**Figure 2:**
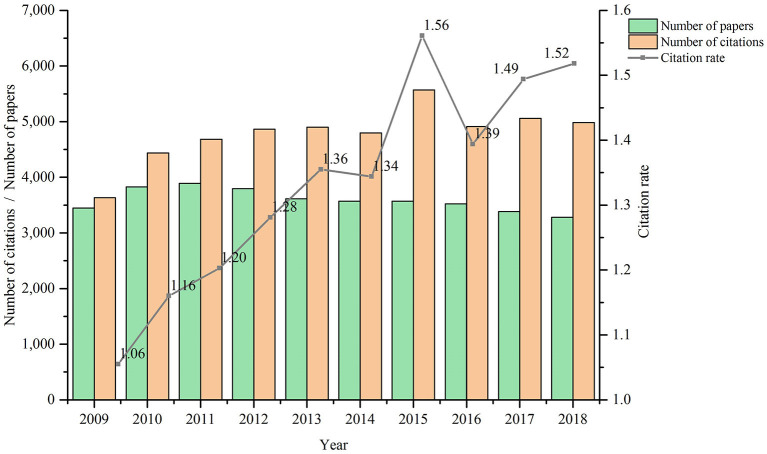
Publication and citation of target papers in the last 10 years.

Horizontally speaking, Chinese scholars prefer to cite English papers. What about the longitudinal situation? We sampled target papers from ScholarCitation from 2009 to 2018, calculated *P*(*ScholarCitation*, [2009, 2018]), *C*(*ScholarCitation*, [2009, 2018]) and *CR*(*ScholarCitation*, [2009, 2018]), and found out the temporal changes of the three indicators. The publication and citation of target papers in the last 10 years are shown in Figure 2.

In terms of the number of papers, it increased first and then decreased from 2009 to 2018. However, the overall fluctuation is modest in that the gap between the maximum and the minimum is within 1,000. In terms of the number of citations, it shows an overall growing trend and reached the maximum in 2015. However, similar to the number of papers, the fluctuation of the number of citations, which remains at around 5,000, is slight. As the result of the calculation of the number of papers and citations, citation rate is a more comprehensive indicator. As is seen in the figure, citation rate shows an overall upward trend and reached a maximum of 1.56 in 2015. This is because the number of papers in each year differs little, but in 2015 the number of citations reached a maximum. Therefore, as a whole, **Chinese papers in the field of computer science show a development trend of “high quality in small amount.”** However, although citation rate is on the rise, there is still a gap between it and self-citation rate (2.60) of the above-mentioned Chinese and English journal papers. This situation is not only aroused by the quality of target papers, but also by Chinese scholars' preference to cite English papers.

### Fields-Based Citation Analysis

#### Publication and Citation

**Figure 3:**
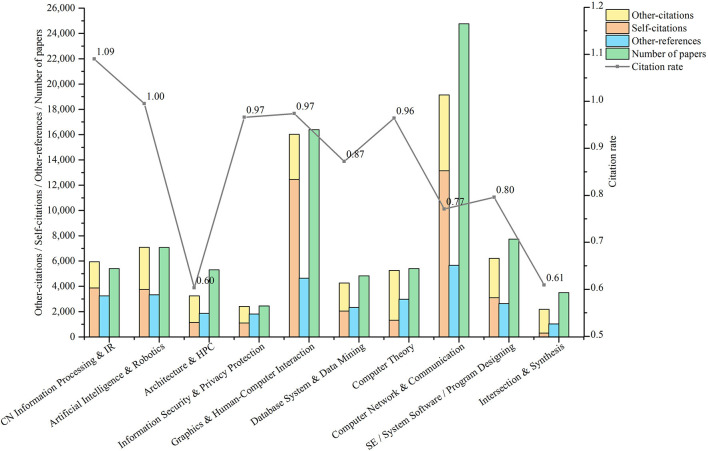
Publication and citation of target papers in 10 fields.

In order to understand the development of different sub-fields of computers, we extracted citation data collected by ScholarCitation from 1960 to 2018 to calculate 5 indicators for each field: the number of papers published (i.e., *P*(*field*, [1960, 2018])), the number of self-citations (i.e., *C*_*S*−*S*_(*field*, [1960, 2018])), the number of other-citations (i.e., *C*_*O*−*S*_(*field*, [1960, 2018])), the number of other-references (i.e., *C*_*S*−*O*_(*field*, [1960, 2018]), and citation rate (i.e., *CedR*(*field*)). The publication and citation of papers in 10 sub-fields of computer are shown in Figure 3. We will analyze it below.

**First, the total number of papers published in each field is positively correlated with the number of citations**. In terms of the number of citations, two most frequently published and cited fields are Network, and Graphics & HCI from 1960 to 2018. The numeric value of the two is way ahead. The two least frequently cited fields are Security and Intersection. In terms of citation rate, the two fields with the highest citation rate are Info and AI. The two fields with the lowest citation rate are Architecture and Intersection. As is seen in the figure above, the total number of papers published in each field is positively correlated with the number of citations (correlation coefficient is 0.98), so the rate of citation in each field, that is, the ratio of the number of citations to the number of papers, fluctuates little (standard deviation is 0.17).

**Second, there are differences in the composition of citations among different fields**. In terms of the composition of citations, the fields where the number of self-citations is significantly higher than that of other-citations are Info, Graphics & HCI, as well as Network. On the other hand, the fields where the number of other-citations is significantly higher than that of self-citations are Architecture, Theory, and Intersection. Therefore, the degree of interdependence among different fields differs.

**Third, there are differences in citation preference among different fields**. In terms of citation preference, the fields of Graphics & HCI, and Network prefer to cite papers in their own fields, while the fields of Theory, Architecture, and Security prefer to cite papers in other fields. This conclusion and the second point above can mutually confirm, indicating that the degree of interdependence among different fields is different.

#### Cumulative Citation

**Figure 4:**
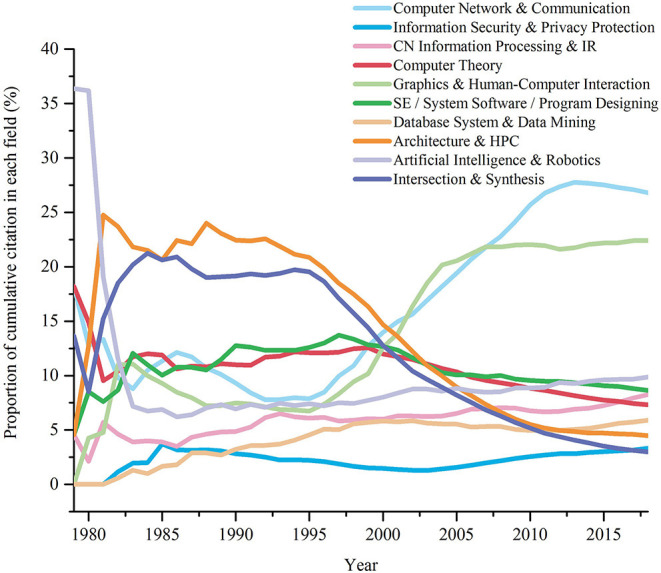
Changes in the proportion of cumulative citation in 10 fields.

Next, we will further analyze the historical development of fields according to the proportion of cumulative citation in different fields. *Cumulative citation* of the target field in a period T is the sum of citations of target papers in this field in T, which is denoted as *Ced*(*field, T*). In order to compare the differences of citations in different fields, we introduce the concept of proportion. *The proportion of cumulative citation* in a target field refers to the ratio of cumulative citation of the target field to that of all fields. Figure 4 is the polyline graph of the proportion of cumulative citation in various fields of computer over time. We will analyze it below.

**First, the development of Architecture is slowing down**. The field of Architecture (HPC) has been the hotspot from 1982 to 2000. But since the twenty-first century, its ranking has been gradually declining: In 2001, it was surpassed by Network, as well as Graphics & HCI, falling to the third place. In 2003, the fields of Theory, Software developed rapidly, rendering a decline in the ranking of HPC to the 5th place. In 2006, 2008 and 2012, the fields of AI, Info, and database and data mining surpassed HPC, respectively, so it ended at the 8th place.

**Second, Graphics & HCI, as well as Network have become hot fields**. The fields of Graphics & HCI, and Network ranked the same for the first time in 1982. After they reached their peaks in 1983 and 1986, respectively, their rankings began to decline slowly. And they got the same rankings again in the 1990s, when they were in their troughs. However, since 1995, the two have grown rapidly and succeeded in occupying the top two places in the twenty-first century.

**Third, Software and Theory are neck and neck**. Although their starting points are sharply different, since 1983, the proportion of cumulative number of citations in Software and Theory has been very close to each other. The former performed slightly worse than the latter from 1983 to 1989, while from 1989 to 2003, the latter was overtaken by the former, but the gap was not huge. The field of Theory gained a weak leading edge again from 2003 to 2006. And from 2006 to 2018, the field of Software was slightly better.

### Journals-Based Citation Analysis

#### Publication and Citation

**Figure 5:**
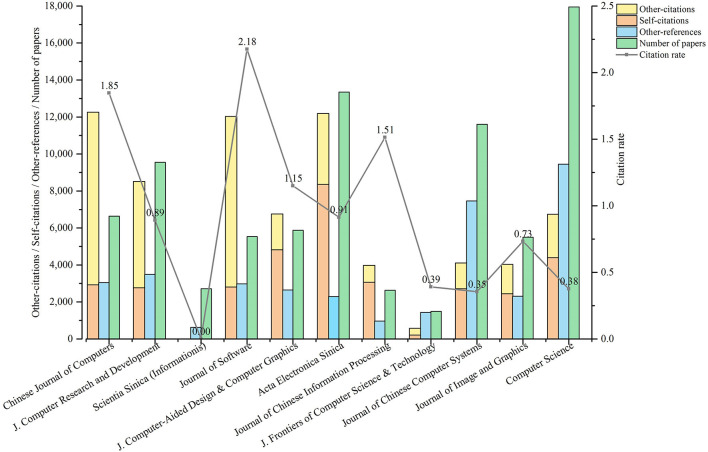
Publication and citation of target papers of 11 most representative journals.

In order to understand the development of different computer journals, we extracted the citation data of ScholarCitation from 1960 to 2018 to calculate 5 indicators for each journal: the number of published papers (i.e., *P*(*journal*, [1960, 2018])), the number of self-citations (i.e., *C*_*S*−*S*_(*journal*, [1960, 2018])), the number of other-citations (i.e., *C*_*O*−*S*_(*journal*, [1960, 2018])), the number of other-references (i.e., *C*_*S*−*O*_(*journal*, [1960, 2018])), and citation rate (i.e., *CedR*(*journal*)). The publication and citation of papers in eleven most representative journals of computer are shown in Figure 5. We will analyze it below.

**First, the citation rate of each journal has little relation with the time it was established**. Due to the different starting time and volume of each journal, the absolute number of papers published and cited cannot be used to measure the influence of journals. Instead, citation rate can reflect the influence of journals to some extent. From 1960 to 2018, the three journals with the highest citation rates are *JS* (founded in 1990), *CJC* (founded in 1978) and *JCIP* (founded in 1986). The lowest are *SS-I* (founded in 1950), *JCCS* (founded in 1980), *CS* (founded in 1974) and *JFCST* (founded in 2007). It can be seen that the citation rate of a journal has little relation with its starting time.

**Second, there are differences in the composition of citations among different journals**. In terms of the composition of citations, the journals with more self-citations than other-citations include *JCAD*&*CG, AES, JCIP, JCCS*, and *CS*. On the contrary, the journals with more other-citations than self-citations include *CJC, JCRD*, and *JS*. From the analysis above, the significance of the three most representative Chinese journals (*CJC, JS*, and *JCRD*) of computer science can be inferred.

**Third, there are differences in citation preference among different journals**. In terms of citation preference, *JCAD*&*CG, AES*, and *JCIP* prefer to cite their own papers, while *JFCST, JCCS*, and *CS* prefer to cite papers from other journals.

#### Cumulative Citation

**Figure 6:**
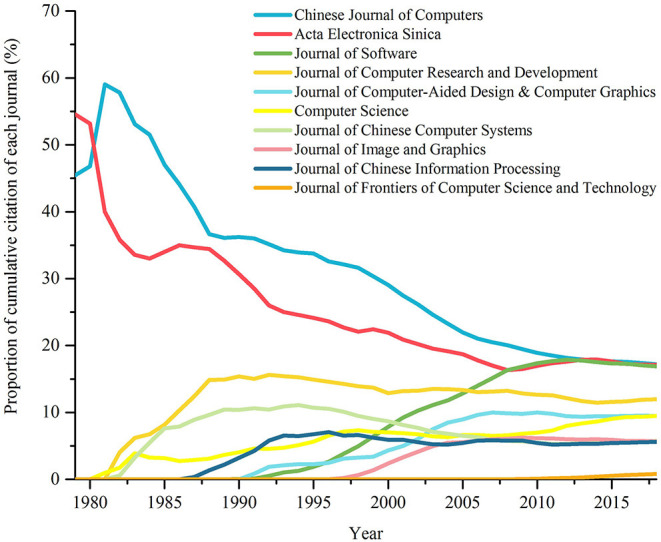
Changes in the proportion of cumulative citation of 11 most representative journals.

In order to further analyze the historical development of each journal, we use the indicator of the proportion of cumulative citation of different journals. *Cumulative citation* of a target journal J in a period T is the sum of citations of target papers published by J in T, which is denoted as *Ced*(*J, T*). In order to compare the differences of citations of different journals, we introduce the concept of proportion. *Proportion of cumulative citation* of a target journal refers to the ratio of cumulative citation of the target journal to that of all journals. Figure 6 is the polyline graph of the proportion of cumulative citation of 10 most representative journals over time. We will analyze it below.

As is shown in Figure 6, by 2018, the top 5 journals in terms of cumulative citation are *Chinese Journal of Computers, AES, JS, JCRD*, and *JCAD*&*CG*. These five outstanding journals fall into two categories: stably developing veterans and rapidly rising stars.

**The first is the stably developing veterans**. From the perspective of rankings, the performance of journals established for a long time are relatively stable. Except being overtaken by *JS* in 2009-2012 and 2016-2017, *CJC* (founded in 1978) and *AES* (founded in 1962), have consistently ranked in the top two. As for *JCRD*, founded in 1982, it held the third place till it was overtaken by *JS* in 2006, and has ranked the fourth since then. However, it is worthy to mention that according to the numeric value of proportion of cumulative citation, *CJC* and *AES* show a downward trend.

**Then there are the rapidly rising stars**. After 10 years' hard work, *JCAD*&*CG* (founded in 1989) and *JS* (founded in 1990), both developed rapidly in the twenty-first century. The former has been in the top five since 2003, and the latter has been in the top three since 2006. *JS* even surpassed *AES*, obtaining the second place.

### Institutions-Based Citation Analysis

#### Cumulative Citation Score

**Figure 7:**
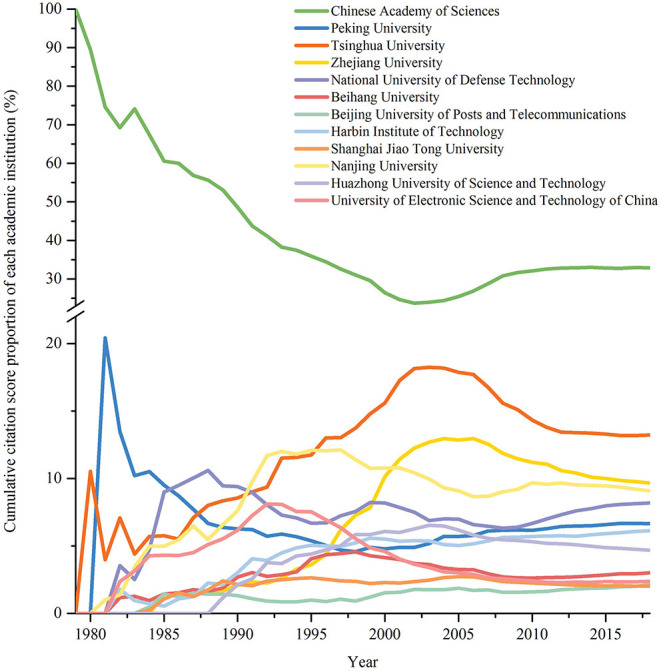
Temporal development of some institutions' cumulative citation score proportions.

As is the case in citation analysis based on fields and journals, in order to analyze the historical development of each institution, the proportion of the cumulative citation score of each institution will be analyzed below. *Cumulative citation score* of a target institution I in a period T is the sum of the citation scores of target papers published by the corresponding institution in T, which is denoted as *CS*(*I, T*). To compare differences between cumulative citation scores of various institutions, we introduce *cumulative citation score proportion*, which refers to the ratio of cumulative citation scores of the target institution to that of all institutions. Figure 7 shows the temporal development of some institutions' cumulative citation score proportions from 1979 to 2018. Except CAS, the selected institutions all received A^+^ or A on Computer Science and Technology in the 4th round of subject evaluation conducted by Ministry of Education of China.

It can be inferred from Figure 7 that **CAS tops the list**. From 1979 to 2018, *cumulative citation score* of CAS has always been the highest one, leaving institutions in the second place far behind. However, **in terms of numerical value, cumulative citation score proportion of CAS fluctuated**, descending from 1979 to 2002, increasing from 2002 and 2008 and achieving stability at around 36% from 2008 to 2018. High ranks of CAS mainly attributes to its sheer size. As a whole, **ranks of institutions have remained stable since the twenty-first century, especially in the last decade**.

#### Average Citation Score

For each institution, we calculated its ranking of average citation score in the last 10 years and compared the rankings with the assessment in the 4th round of subject evaluation conducted by Ministry of Education of China on Computer Science and Technology^9^ As is announced, numbers of institutions receiving A^+^, A, A^−^, B^+^, B, C^+^ are 4, 7, 12, 24, 24, 26, respectively. An institution is *accurate-evaluated* if it receives an A^+^(A, A^−^, B^+^, B, C^+^) in the assessment conducted by Ministry of Education, and ranks No.1~No.4(No.5~No.11, No.12~No.23, No.24~No.47, No.48~No.71, No.72~No.97) after our calculation. We calculated the accuracy of each rating according to the definition above.

**Figure 8:**
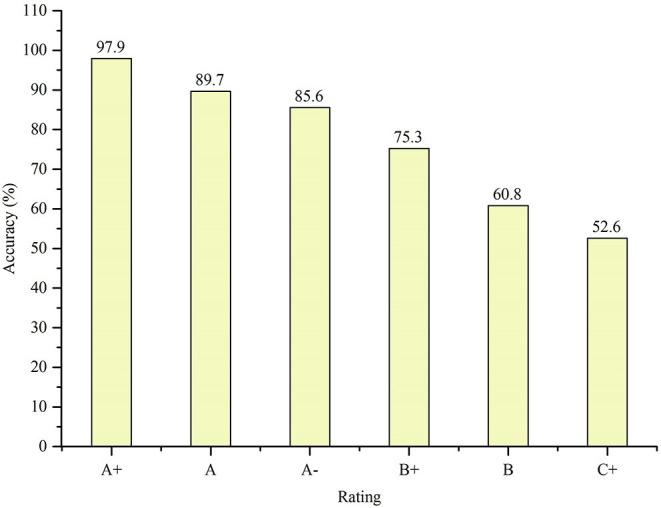
Accuracy of each rating.

Figure 8 shows the accuracy of each rating. It can be inferred from the table that the ranking results of institutions' average citation scores in recent 10 years **are consistent with** the evaluation results of Ministry of Education of China.

### Cross Citation Analysis

**Figure 9:**
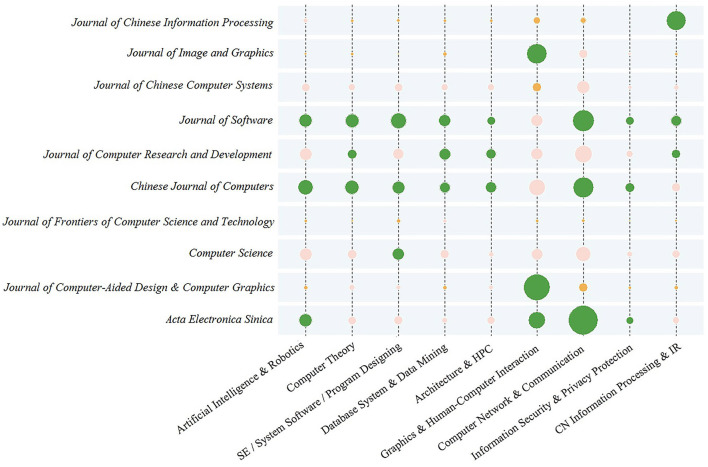
Number of citation of journals in certain fields.

Cross citation analysis is based on journal-field. Generally speaking, in terms of the subject of computer science, papers collected by English journals are focused on a certain field, while Chinese journals tend to be more comprehensive. That is to say, a journal may include target papers from multiple fields simultaneously. Similarly, target papers in a particular field may be distributed among different journals. Figure 9 takes the field as the horizontal coordinate and the journal as the vertical coordinate, and presents the number of citations of each journal in the corresponding field from 1960 to 2018 in the form of matrix bubble graph. Except *SS-I*, target journals are all included in Figure 9. The larger the bubble area is, the more citations the journal receives in the corresponding field. Bubble color indicates the rankings: green (orange) indicates that the corresponding journal is among the top (last) 3, considering number of citations in the corresponding field; Pink indicates other middle places.

From the analysis of Figure 9, the following conclusions can be drawn. Firstly, three **most representative Chinese journals of computer science present a situation of tripartite confrontation and are typical comprehensive journals**. As a consensus, three most representative Chinese journals of computer science are *CJC, JS*, and *JCRD*. They show strong performance, occupying at least two of the top three positions in all fields except Graphics & HCI. Secondly, **journals oriented to specific fields to some extent represent the authority of their own fields and are typical characteristic journals**. For instance, *JIG* and *JCAD*&*CG* win the top two in the field of Graphics & HCI, while *JCIP* is the leader in the field of Info.

## Conclusion

Based on citation relationships collected in ScholarCitation system, we conduct a citation analysis of Chinese journals in the field of computer science with a time span of 60 years. As a whole, Chinese papers in the field of computer science shows a trend of “high quality in small amount.” The number of papers increased and then decreased in the last decade. However, the number of citations showed an overall growing trend. Merely considering self-citation, Chinese scholars tend to cite papers in English.

Three most representative Chinese journals of computer science present a situation of tripartite confrontation and are typical comprehensive journals. They show high coincidence of cooperative institutions and cooperate closely with multi-cooperation institutions. As for characteristic journals, they to some extent represent the authority of their own fields and pay more attention to the dominant institutions in their own fields.

Development trends of different fields and institutions differ. The development of Architecture slowed, while Graphics & HCI, and Network developed into hot fields. In terms of institutions, ranks of institutions have remained stable since the twenty-first century, especially in the last decade.

Journals, institutions and scholars should strengthen their cooperation, working to improve the quality of papers together. More analysis should also be conducted on literature in non-English languages to boost diversity.

## Data Availability Statement

The datasets generated for this study are available on request to the corresponding author.

## Author Contributions

All authors listed have made a substantial, direct and intellectual contribution to the work, and approved it for publication.

### Conflict of Interest

The authors declare that the research was conducted in the absence of any commercial or financial relationships that could be construed as a potential conflict of interest.

## Footnotes

^1^https://dblp.uni-trier.de/

^2^https://www.scopus.com/

^3^http://apps.webofknowledge.com/

^4^https://academic.microsoft.com/

^5^http://csrankings.org/

^6^http://cdblp.ruc.edu.cn/

^7^http://csrankings.org/

^8^https://www.scimagojr.com/countryrank.php?category=1701

^9^http://www.cdgdc.edu.cn/xwyyjsjyxx/xkpgjg/index.shtml
